# Treatment of Sick Headache

**Published:** 1873-03

**Authors:** 


### ARTICLE VI.

### *Treatment of Sick Headache.*

About a year ago there was published in the *British Medical Journal* the results of the experience of a large number of leading British physicians regarding the treatment of this distressing affection. The substance of the report amounted pretty much to this: that the relief afforded by the medical man was, as a rule, so trifling that the patient took the matter into his own hands, and found by experience the best way to obviate or alleviate the attack, or made up his mind that the affection was incurable. Bromine of potassium was, however, noticed as a drug occasionally capable of affording relief. Dr. Williams, of the Sussex Lunatic Asylum, also recorded the experience, at that institution, of Indian hemp as a remedy, and stated the opinion of his colleague Dr. Greene and himself, that this drug was worthy of extended trial. Since the appearance of our report, guarana or paullinia powder, a remedy for sick headache which has been employed in France and elsewhere for some years, was brought prominently before the notice of the profession in this country by Dr. Wilks. * * "The results obtained are very various, and show that our knowledge of its *modus operandi* is most incomplete. An extended trial of guarana, and a careful observation of the class of cases in which it is of value, will probably ere long lead to a more definite knowledge of its therapeutical value. We are glad to have the opportunity of recording the views of Dr. Wilks on guarana and the other remedies which his special attention to the subject has led him to employ since the publication of his first paper. The opinion entertained by him regarding the value of cannabis Indica is shared by another of the writers in the report. Indian hemp seems deserving of a full trial.

"As regards the treatment of sick headache," says Dr. Wilks, "I have until late years been able to do little more than recommend to my patients the avoidance of all those circumstances which they know from experience would induce an attack. I allude, of course, to nervous headache, for I believe that this is almost the only form for which we are consulted; a temporary disturbance of the head arising from various causes, requiring no medicine, or but a casual dose. During the last three or four years I have been able, with others in my profession, to do something more positive than preach hygienic principles to my patients, having in our profession three remedies which have been doing eminent service in this terrible complaint. In the first place there is the bromide of potassium, which is so valuable a medicine in many cases of sick headache that it can scarcely be superseded by a better remedy. The patient, who is very often a gentleman, comes home with a splitting headache, fatigued and worried after a hard day's work; he takes fifteen or twenty grains of bromide of potassium, presently goes off to sleep in his easy chair, and wakes in an hour well. I have known this to occur in so many instances, that I cannot hesitate in my belief as to the efficacy of this medicine. It is one which I always first employ, having seen such eminent advantages follow its use. I have known many patients declare that the bromide was the first medicine they had taken in their lives which had the slightest effect in relieving their headache.

"About two years ago I commenced to use the cannabis indica, and I have no hesitancy in saying that in this drug we possess a most valuable remedy against headache. I have never given it in large doses with the object of counteracting the pain by producing an immediate effect, but have employed it in doses of a few drops three times a day, and continued for some weeks. In several cases where my patients were subjected to constant headache, great benefit was experienced, several of them having written to say that they had got rid of their trouble, or it had been less frequent. I consider it superior to all other remedies in this respect, that, it efficacous at all, it preserves the patient from his malady; whereas other medicines do little more than arrest the attacks when they have commenced. I have not given cannabis in the manner recommended by Dr. Williams (of *Hayward's Health*) in the form of a dose of the extract daily.

"Thirdly, guarana had been introduced to our notice as a remedy for sick headache, and here, again, we have a very valuable addition to our *Phamacopœia.* In many instances, especially those of ladies, I have had the most positive assurance given me of the power of this drug in arresting headache, so that not the slightest doubt can be entertained of its immense value. A dose is usually taken when the headache is approaching; and if this is not quickly successful in arresting it, a second powder is swallowed; after an hour or so, if the remedy is to be useful, the headache has disappeared. I know of several cases in which the greatest enthusiasm is expressed by patients as to its merits. At the same time I am constantly hearing of cases where it has failed. I am now trying it in smaller doses by daily administration.

"I feel certain that these three drugs—bromide of potassium, cannabis Indicas and guarana—constitute a most important addition to our nervine remedies against a terrible complaint, which, a few years ago, constituted the opprobrium of medicine. I might say that I know of cases where galvanism had very speedily cured a pain in the head, and I can call to mind the case of a lady where the application of the bisulphide of carbon invariably relieved the most severe headaches."—*British Medical Journal.*

